# Correlation Between Pretreatment Neutrophil-to-Lymphocyte Ratio and Programmed Death-Ligand 1 Expression as Prognostic Markers in Non-Small Cell Lung Cancer

**DOI:** 10.7759/cureus.26843

**Published:** 2022-07-14

**Authors:** Cristina-Florina Pirlog, Horia Teodor Cotan, Andreea Parosanu, Cristina Orlov Slavu, Ana Maria Popa, Cristian Iaciu, Mihaela Olaru, Alexandru Vlad Oprita, Irina Nita, Cornelia Nitipir

**Affiliations:** 1 Oncology, Elias Emergency University Hospital, Bucharest, ROU; 2 Medical Oncology, Carol Davila University of Medicine and Pharmacy, Bucharest, ROU; 3 Faculty of Medicine, Carol Davila University of Medicine and Pharmacy, Bucharest, ROU; 4 Medical Oncology, Elias Emergency University Hospital, Bucharest, ROU

**Keywords:** survival time, systemic inflammation index, nlr, pd-l1 expression, lung cancer

## Abstract

Background

The neutrophil-to-lymphocyte ratio (NLR) at baseline treatment is an important marker of systemic inflammation, which is correlated with survival benefits in lung, breast, ovarian, bladder, and colorectal cancer. Programmed death-ligand 1 (PD-L1) expression is a biomarker with discording results regarding survival benefits in lung cancer. In our research, we studied the relationship between these two markers in patients with lung cancer.

Methods

Patients with stage I, II, III, and IV lung cancer (n = 80) were included in this retrospective study. The NLR baseline was recorded before the initiation of treatment. The NLR cut-off value was 4. PD-L1 expression was determined by immunohistochemical staining. Univariate and multivariate survival analyses were conducted to test their prognostic value.

Results

NLR proved to be a significant prognostic factor for progression-free survival (PFS) (p=0.002, Log Rank) with a mean PFS of 27.7 months for low NLR patients and 12.8 months for high NLR patients. It was also significant for overall survival (OS) (p=0.007, Log Rank) with a mean OS of 52 months for low NLR patients and 41.6 months for high NLR patients. The prognostic impact of PD-L1 expression on PFS and OS was not statistically significant with a mean PFS of 23.1 months for PD-L1-negative patients and 15.8 months for PD-L1-positive patients (p=0.422, Log Rank). Mean OS was 49 months for PD-L1-negative patients while for PD-L1-positive patients, it was 43.3 months (p=0.550 Log Rank). Regarding the correlation between PD-L1 expression and NLR value, PFS mean survival times were 13.1 months for PD-L1(+)/NLR>4, 15.1 months for PD-L1(-)/NLR>4, 16.4 months for PD-L1(+)/NLR<4 and 27.8 months for PD-L1(-)/NLR<4. This correlation between PFS and the combined PD-L1 and NLR prognostic factor was statistically relevant (p=0.04). For OS, the PD-L1/NLR combined prognostic factor was not statistically relevant (p=0.055). A mean PFS time of 27.8 months was reported for PD-L1(-)/NLR<4 group patients while for the other groups, the mean PFS was 14.9 months (p=0.045). In univariate analysis, the elevated NLR was significantly associated with a decreased PFS time (HR=2.31, 95% CI =1.323- 4.051, p=0.03) as well as OS (HR=3.555, 95% CI=1.310- 9.652, p=0.013). In multivariate analysis, NLR remained statistically significant for PFS (HR=2.160, 95% CI=1.148- 4.062, p=0.013) and OS (HR=4.364, 95% CI=1.474- 12.921, p=0.008) after adjusting for the factors of age, gender, tumor stage, lymph node stage, clinical stage, histology, and PD-L1 expression. PD-L1 expression was not a valid prognostic factor for progression or death in either univariate or multivariate analysis. We also stratified the disease control rate (DCR) depending on PD-L1/NLR combined factor expression. In the PD-L1(-)/NLR<4 group, we had the highest number of partial responses (PRs) and only one complete response (CR) compared to the other groups (p=0.006).

Conclusions

As the number of patients is limited in the present analysis, it is hypothesized that these two markers can be useful in dividing patients into two prognostic groups: the good prognostic group reunites PD-L1(+)/NLR<4 and PD-L1(-)/NLR<4 and the poor prognostic group reunites PD-L1(+)/NLR>4 and PD-L1(-)/NLR>4.

## Introduction

Lung cancer (LC) is the second most frequent cancer with an incidence rate of 11.4% and the first cause of death of all cancers with a mortality rate of 18% [1]. The five-year survival rate is ranging between 6% and 19%, even though there are new modalities of diagnosis and treatment. The most frequent subtype of LC is non-small cell lung cancer (NSCLC), which accounts for 80-85% of cases. The other subtype is small cell lung cancer (SCLC), which accounts for 15% of cases and the survival rate at five years reaches only 6%. The latter is known as being the most aggressive LC [2].

In recent years, immunotherapy with or without chemotherapy has become the main treatment for LC. Even with immunotherapy survival time in metastatic NSCLC is on average 10-12 months, although for patients with programmed death-ligand 1 (PD-L1) positive status, survival reaches 20 months [3]. Latest studies are concentrating on finding biomarkers for a better selection of patients with LC that respond to immunotherapy.

The first biomarker proposed for these patients was PD-L1 expression. Programmed cell death protein 1 (PD-1) is a protein expressed by T and B-activated cells, which is involved in regulating the immune response. It has two ligands (PD-L1 and PD-L2), of which PD-L1 has a variable expression in tumor cells such as those present in LC [4]. PD-L1 expression was validated in Keynote-001, patients with high expression (>50%) had a better progression-free survival (PFS) and overall survival (OS) than those with low expression [5-6]. There are also some studies with discordant results regarding PD-L1 expression, some of them suggesting there is no connection between survival and PD-L1 expression [7] with others suggesting that PD-L1 status is a negative prognostic factor [4,8-9].

Several studies have shown that systemic inflammation plays an important role in tumor development and progression. Systemic inflammation is also responsible for resistance to classic treatments [10-13]. Neutrophil to lymphocyte ratio (NLR) is a parameter that quantifies systemic inflammation, and it was identified as an independent prognostic factor in numerous malignancies. Recent studies have proposed baseline NLR as a prognostic marker for LC [4,13-15].

Our study demonstrated a link between PD-L1 status and NLR as a marker of systemic inflammation.

## Materials and methods

Patients

A total of 80 patients with histologically confirmed NSCLC were included in this retrospective single-center study, conducted at the Department of Oncology, Elias Emergency Hospital Bucharest between 2016 and 2021. The follow-up time varied between four months and 72 months with an average follow-up time of 21 months.

Inclusion criteria consisted of a positive histopathological diagnosis of NSCLC obtained through direct tumor biopsy, regional adenopathies, or distant metastases that were accessible for biopsy, as well as an accurate staging according to the American Joint Committee on Cancer (AJCC) criteria. Sufficient tumor material for immunohistochemical analysis of PD-L1 expression was also necessary. Patients also had a blood sample analyzed for CBC at most three days before initiation of treatment. Exclusion criteria consisted of any signs or symptoms of infection such as elevated procalcitonin, leukocytosis, fever, malaise, abnormal chest radiography, positive blood culture, urine culture, and pharyngeal exudate, which would modify the NLR. Patients with immunocompromised status or other autoimmune diseases were excluded as well. Also, we excluded patients treated with immunosuppressives, immunomodulators, or steroids. The absence of a PD-L1 score was also an exclusion criterion. All patients signed informed consent.

Pretreatment NLR was defined as the absolute count of neutrophils divided by the absolute count of lymphocytes, measured before the initiation of oncologic treatment. PD-L1 was evaluated with immunohistochemistry. The percentage of tumor cells with membrane staining for PD-L1 (TPS) was recorded. TPS was calculated using the total number of PD-L1-positive tumor cells divided by the total number of PD-L1-positive and negative tumor cells multiplied by 100. NLR was calculated by dividing the absolute number of neutrophils by the absolute number of lymphocytes.

We mention that the absolute count of neutrophils was calculated using the formula WBC (white blood count) x total neutrophils (segmented neutrophils % + segmented bands %) x 10 while the absolute lymphocyte count was calculated by multiplying the total number of white blood cells against the percentage of white blood cells, which are lymphocytes.

Immunohistochemical staining

Sections from formalin-fixed paraffin-embedded tissue blocks were deparaffinized and treated using standard procedures. Antibodies for PD-L1 were used based on the manufacturer's instructions. There were different clones used for PD-L1 assessment: some patients were tested with Dako 22C3; for others, Ventana SP263 was used.

Immunohistochemical evaluation

PD-L1 expression was defined by TPS. A score lower than <1% was considered negative. The positive expression was classified into low expression (TPS = 1-49%) and high expression (TPS ≥ 50%).

Statistical analysis

The data were analyzed using SPSS software V.26 (IBM Corp., Armonk, NY). The end goal of the study was to determine the correlation between NLR and PD-L1 as well as the statistical relevance of NLR, PD-L1, and the combined PD-L1/NLR as prognostic markers for PFS and OS. The correlation between these factors and PFS as well as OS was analyzed using Kaplan-Meier curves and Cox-regression analysis, with the latter adjusted for age, gender, T-stage, N-stage, and clinical stage. Spearman’s rho test was used to explore the association between PD-L1 and NLR. A ROC curve was used to determine the optimal cut-off value for NLR. PFS was defined as the time from initiation of treatment to progression while OS was defined as the time from diagnosis until patient’s death. Results were considered significant when the p-value was less than 0.05.

## Results

The descriptive statistics of the patient’s characteristics and the association with PFS and OS are shown in Tables 1-3.

**Table 1:** Patient characteristics NLR: Neutrophil to Lymphocyte Ratio; PD-L1: Programmed Death-Ligand 1; EGFR: Epidermal Growth Factor Receptor; ALK: Anaplastic Lymphoma Kinase; NTRK: Neurotrophic Tyrosine Receptor Kinase

	N	%
Age (median) Gender	66 years (44- 88)	
	Male		52	65
Female	28	35
Histology		
Adenocarcinoma	59	73.8
Squamous	19	23.8
Other	2	2.5
T stage		
T1	9	11.3
T2	11	8.9
T3	69	56.1
T4	43	35
N lymph nodes		
N0	8	10
N1	15	18.8
N2	29	36.3
N3	28	35
PD-L1 expression		
<1%	38	47.5
1-49%	19	23.8
>50%	23	28.8
Stage at diagnosis		
I	1	1.3
II	5	6.3
III	20	25
IV	54	67.5
NLR		
< 4	40	50
>4	40	50
PD-L1/NLR		
PD-L1(+)/NLR<4	13	16.3
PD-L1(+)/NLR>4	27	33.8
PD-L1(-)/NLR<4	25	31.3
PD-L1(-)/NLR>4	15	18.8
Driver mutations		
EGFR	12	75
ALK	3	18.75
NTRK	1	6.25

**Table 2:** Association of clinicopathological features with PFS SCC: Squamous Cell Carcinoma; ADC: Adenocarcinoma; NLR: Neutrophil to Lymphocyte Ratio; PD-L1: Programmed Death-Ligand 1; PFS: Progression-Free Survival

		PFS	
		HR	CI	p-Value
Age (<60 vs. >60)	1.264	0.692- 2.310	0.446
Gender (male vs. female)	0.840	0.545- 1.292	0.427
T stage (1/2 vs. 3/4)	1.274	0.719- 2.255	0.406
N stage (0/1 vs. 2/3)	1.730	0.906- 3.302	0.084
Histology (SCC/other vs. ADC)	1.124	0.686- 1.435	0.966
Stage (I/II vs. III/IV)	1.368	0.539- 3.468	0.019
PD-L1 expression (negative vs positive)	1.244	0.718- 2.153	0.435
NLR	2.160	1.148- 4.062	0.013
Other vs PD-L1(-)/NLR<4	0.557	0.299- 1.038	0.050

**Table 3:** Association of clinicopathological features with OS SCC: Squamous Cell Carcinoma; ADC: Adenocarcinoma; NLR: Neutrophil to Lymphocyte Ratio; PD-L1- Programmed Death-Ligand 1; OS: Overall Survival

		OS	
		HR	CI	p-Value
Age (<60 vs. >60)	1.653	0.610- 4.477	0.610
Gender (male vs. female)	0.812	0.324- 2.033	0.427
T stage (1/2 vs. 3/4)	1.213	0.442- 3.326	0.707
N stage (0/1 vs. 2/3)	1.135	0.427- 3.016	0.800
Histology (SCC/other vs. ADC)	1.076	0.331- 2.112	0.705
Stage (I/II vs. III/IV)	1.051	0.097- 11.350	0.050
PD-L1 expression (negative vs positive)	1.120	0.192- 3.378	0.766
NLR	4.143	1.245- 13.791	0.021
Other vs PD-L1(-)/NLR<4	0.557	0.299- 1.038	0.055

**Figure 1:**
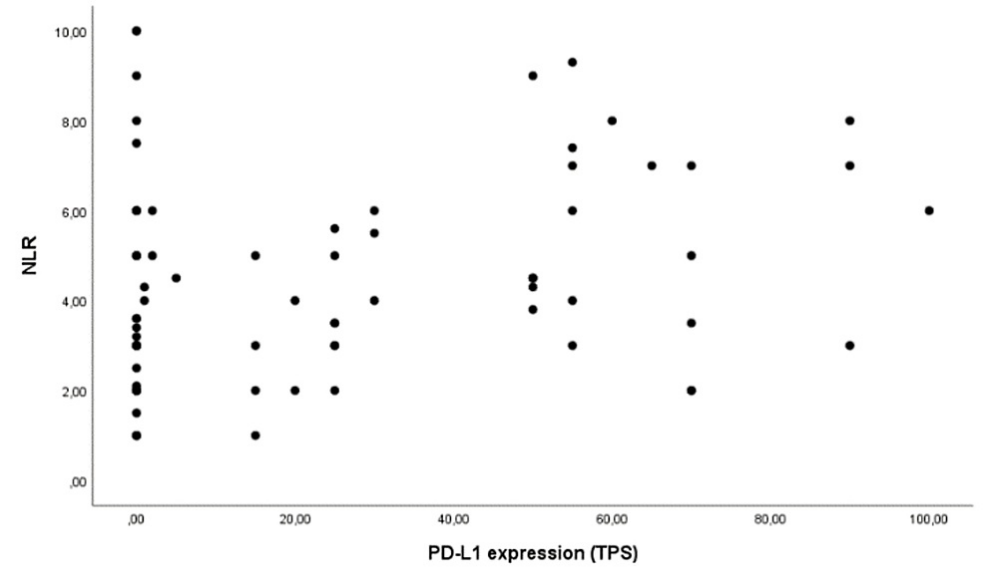
Scatter plot graph representing the distribution of neutrophil-to-lymphocyte ratio (NLR) and PD-L1 expression (TPS)

The distribution of PD-L1 expression according to NLR was represented using a scatter plot graph in Figure 1. There was a weak but significant correlation between NLR and PD-L1 expression (Spearman’s rank correlation ρ2=0.289, p=0.003).

**Figure 2:**
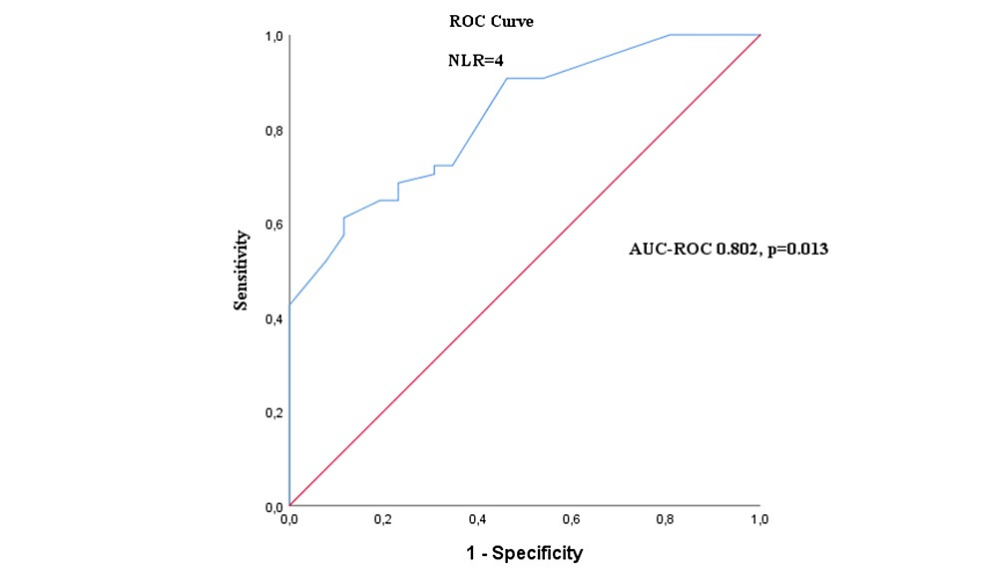
Receiver operating characteristics (ROC) curves used to determine the cut-off value and diagnostic performance of NLR AUC-ROC: Area Under the ROC Curve

The ROC curve proved that NLR was an acceptable and significant factor of prognosis to predict PFS and OS (AUC-ROC, 0.802, p=0.013) (Figure 2). Based on the ROC curve, the median value of 4 was used to classify the patients into high NLR (> 4) and low NLR (< 4). The cut-off value for TPS is 1% with values lower than 1% being considered negative and values higher than 1% being considered positive.

**Figure 3:**
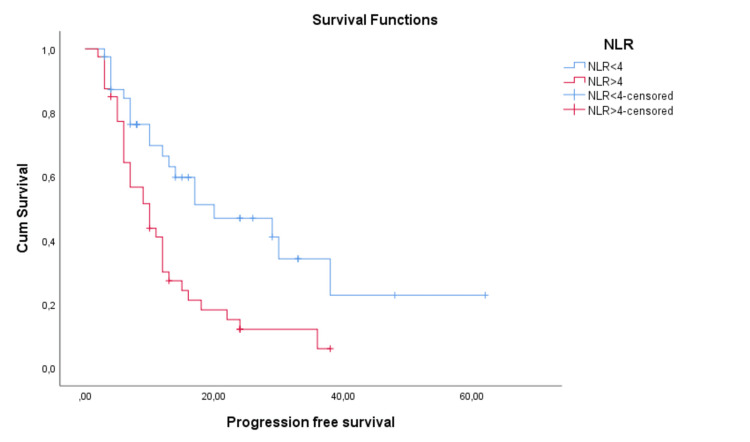
Progression-free survival (PFS) depending on the neutrophil-to-lymphocyte ratio (NLR)

**Figure 4:**
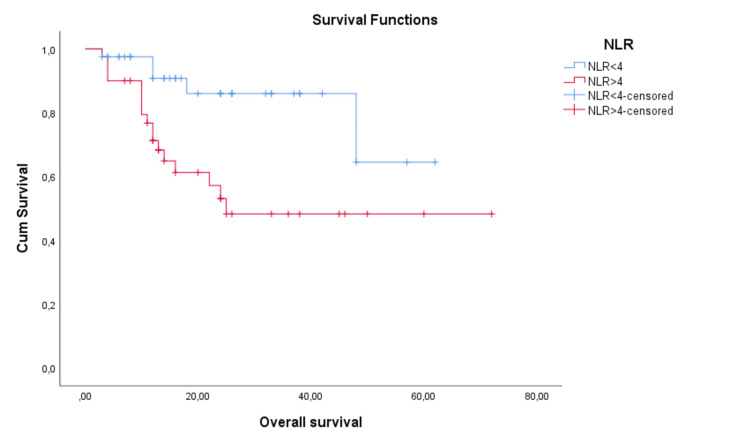
Overall survival (OS) depending on the neutrophil-to-lymphocyte ratio (NLR)

Mean PFS and OS times according to NLR status were represented in Figures 3-4. NLR proved to be a significant prognostic factor for PFS (p=0.002, Log Rank) with a mean PFS of 27.7 months for low NLR patients and 12.8 months for high NLR patients. It was also significant for OS (p=0.007, Log Rank) with a mean OS of 52 months for low NLR patients and 41.6 months for high NLR patients. We have also evaluated median PFS time, with a median PFS of 10.5 months for high NLR patients and 20 months for low NLR patients. We could not evaluate the median OS time because the median was not reached (Figures 3-4).

**Figure 5:**
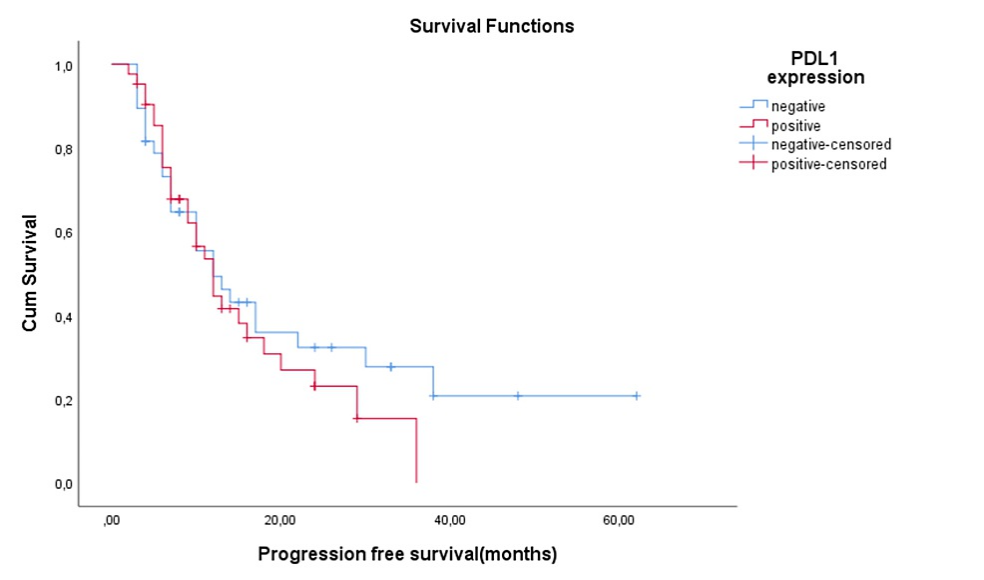
Progression-free survival (PFS) depending on PD-L1 expression (TPS)

**Figure 6:**
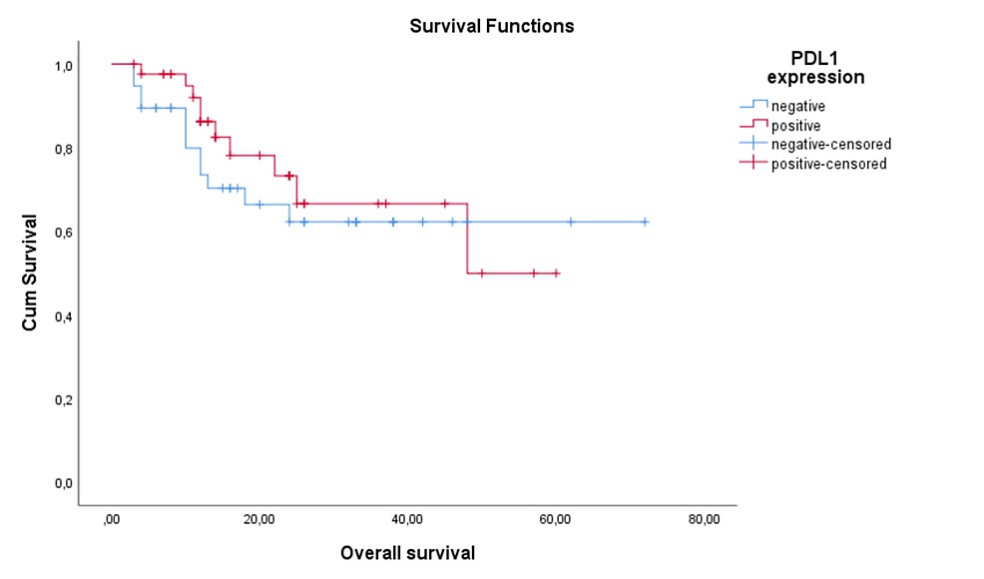
Overall survival depending on PD-L1 expression (TPS)

The prognostic impact of PD-L1 expression on PFS and OS is not statistically significant, with a mean PFS of 23.1 months for PD-L1-negative patients and 15.8 months for PD-L1-positive patients (p=0.422, Log Rank). Median PFS time was not different between the two groups, with a value of 12 months for both PD-L1-positive as well as PD-L1-negative patients. Mean OS time was 49 months for PD-L1-negative patients, while for PD-L1-positive patients, it was 43.3 months (p=0.550, Log Rank). Median OS time was not calculated, as the PD-L1-negative group had not reached the median (Figures 5-6).

**Figure 7:**
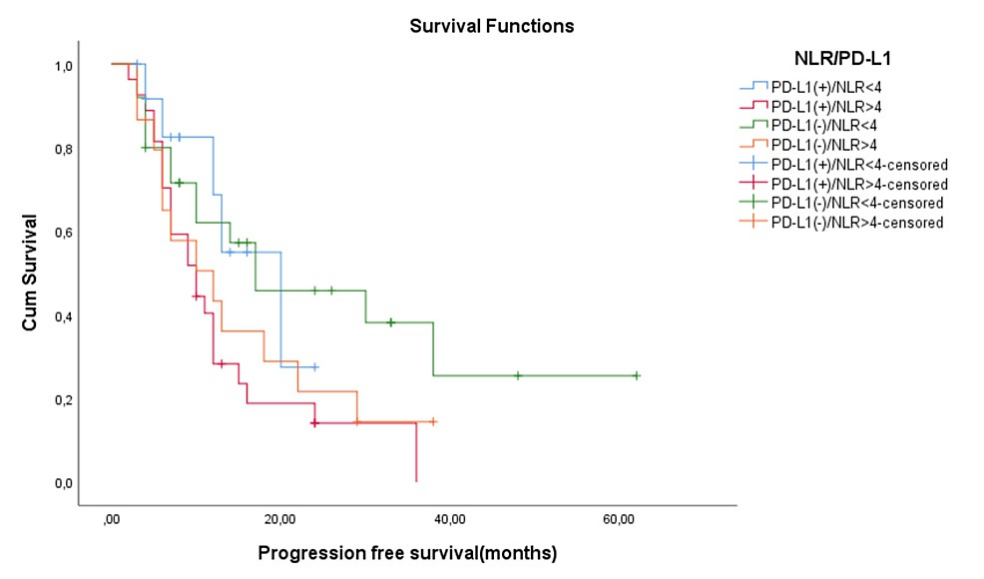
Progression-free survival (PFS) according to tumor proportion score (TPS) for tumoral PD-L1 expression status in combination with the neutrophil-to-lymphocyte ratio (NLR)

We decided to evaluate the prognostic impact of PD-L1 expression on PFS and OS stratified depending on NLR values. We obtained four subgroups: PD-L1(+)/NLR high, PD-L1(+)/NLR low, PD-L1(-)/NLR high, and PD-L1(-)/NLR low. For PFS, the mean survival times were 13.1 months for PD-L1(+)/NLR>4, 15.1 months for PD-L1(-)/NLR>4, 16.4 months for PD-L1(+)/NLR<4, and 27.8 months for PD-L1(-)/NLR<4. As for median PFS time, it was for the PD-L1(+)/NLR>4 group was 10.4 months, for the PD-L1(-)/NLR>4 group, it was 12 months, for the PD-L1(+)/NLR<4 group, it was 20 months, and for the PD-L1(-)/NLR<4 group, it was 17.5 months. This correlation between PFS and the combined PD-L1 and NLR prognostic factor was statistically relevant (p=0.04). For OS, the PD-L1/NLR combined prognostic factor was not statistically relevant (p=0.055) (Figure 7).

**Figure 8:**
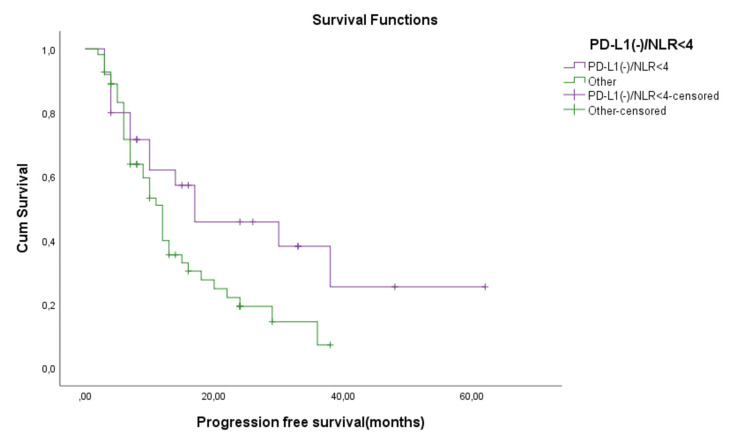
Comparison between PD-L1(-)/NLR<4 and the other groups

A comparison between PD-L1(-)/NLR<4 and the other groups is displayed in Figure 8. A mean PFS time of 27.8 months was reported for PD-L1(-)/NLR<4 group patients while for the other groups, the mean PFS was 14.9 months (p=0.045). The median PFS time for the PD-L1(-)/NLR<4 group was 17 months as compared to the other group, in which the median PFS time was 12 months.

In univariate analysis, the elevated NLR was significantly associated with a decreased PFS time (HR=2.31, 95% CI =1.323-4.051, p=0.03) as well as OS (HR=3.555, 95% CI=1.310-9.652, p=0.013). In multivariate analysis, NLR remained statistically significant for PFS (HR=2.160, 95% CI=1.148- 4.062, p=0.013) and OS (HR=4.364, 95% CI=1.474- 12.921, p=0.008) after adjusting for the factors of age, gender, tumor stage, lymph node stage, clinical stage, histology, and PD-L1 expression. PD-L1 expression was not a valid prognostic factor for progression or death in either univariate or multivariate analysis.

We also stratified the disease control rate (DCR) depending on PD-L1/NLR combined factor expression; the results are shown in Table 4. The PD-L1(-)/NLR<4 group had the highest number of partial responses (PRs) and only one complete response (CR) compared to the other group (p=0.006).

**Table 4:** Disease control rate depending on the PD-L1/NLR combined factor PD: Progressive Disease; SD: Stable Disease; PR: Partial Response; CR: Complete Response; NLR: Neutrophil to Lymphocyte Ratio; PD-L1: Programmed Death-Ligand 1

		PD-L1(+)/NLR<4	PD-L1(+)/NLR>4	PD-L1(-)/NLR<4	PD-L1(-)/NLR>4	p-value
	PD	5	24	15	13	0.006
Disease control rate	SD	8	3	6	1	
	PR	0	0	3	1	
	CR	0	0	1	0	
Total		13	27	25	15	

## Discussion

In this study, we aimed to see if NLR and PDL1 expression are prognostic biomarkers for LC. NLR is regarded as a parameter for systemic inflammation. Acute inflammation conducts to the maturation of dendritic cells and antigen-presenting cells, inducing an anti-tumoral response; in contrast, chronic inflammation leads to tumor progression by activating the NF-κB pathway. The proportion of NLR is driven by neutrophil and lymphocyte count. A high NLR is consistent with a weak immune response directed against the tumor. This poor response may be caused by the tumors' capacity to inhibit the immune response by downregulating T cells [16-17].

Faget et al. reported that tumor-associated neutrophils play a meaningful role in the tumor microenvironment by inhibiting the function of T cells and, at the same time, engaging T reg immunosuppressive cells by modifying the functions of dendritic and macrophage cells, by stimulating neoangiogenesis, by infiltration in the tumor and the reduction of host immune cells, and by the intrinsic characteristics of cancer [18]. Initial systemic inflammation caused by the tumor before the initiation of treatment conducts to poor treatment response rates as well as a reduced duration of response. An elevated NLR baseline is associated with reduced PFS and a poor response to treatment [19].

Other research studies demonstrated a similar effect of NLR on the clinical outcome; a low NLR is consistent with a good prognosis. Patients with NLR < 4 have a longer PFS and OS [14-15,19-20].

In the last years, researchers concentrated their efforts on finding biomarkers that predict good and durable responses to the newest treatments such as PD-L1 expression. Assessment of PD-L1 expression in clinical trials was made with different assays. Every immune checkpoint inhibitor has a companion test, which evaluates the presence of tumor cells, and, some of them, the existence of inflammatory cells [5]. There are different opinions regarding PD-L1 expression; some authors consider it a predictor of a better outcome but only in the early stage of the disease [21]; others consider that its expression is associated with more aggressive characteristics, such as increased tumor size, lymphovascular and perineural invasion, and positive lymph nodes, and, consequently, with a poor prognostic in advanced stages of LC [8-9]. In our study, patients with PD-L1-negative expression tend to have a longer OS and PFS but without statistical significance.

We also evaluated the relationship between NLR and PD-L1 expression. We found that patients with NLR<4 and PD-L1-negative expression have significantly longer PFS but not OS. Likewise, DCR was significant for patients with NLR<4 and PD-L1-negative expression. A possible explanation for this result is that we included more patients with EGFR wild type (only 12 patients with EGFR mutant). Wang et al. showed in their study that the value of the combination between PD-L1/NLR is prognostic only in patients with EGFR wild-type [10]. We know from the literature that patients with EGFR mutation have a high expression of PD-L1, in contrast to EGFR wild-type in which PD-L1 expression tends to be low [22-24]. Another explication could be the PD-L1 assessment with two different clones, although studies showed that there is a high correlation between the results obtained with clone 22C3 by Dako and SP263 by Ventana [25].

Our study has some limitations: first, the limited number of patients; second, the retrospective design; third, the limited number of patients with EGFR mutation status; fourth, the PD-L1 assessment done by two different clones. Our findings need to be evaluated in larger prospective studies.

## Conclusions

In this study, we found that patients with NLR<4 at baseline treatment have a better outcome translated in PFS and OS. PD-L1 expression was not correlated with PFS or OS.

As the number of patients is limited in the present analysis, it is hypothesized that these two markers can be useful in dividing patients into two prognostic groups: the good prognostic group reunites PD-L1(+)/NLR<4 and PD-L1(-)/NLR<4, and the poor prognostic group reunites PD-L1(+)/NLR>4 and PD-L1(-)/NLR>4. Patients with a PD-L1-negative expression and NLR<4 had a better DCR than other groups.
